# An interpretive study of food, snack and beverage advertisements in rural and urban El Salvador

**DOI:** 10.1186/s12889-015-1836-9

**Published:** 2015-05-30

**Authors:** Baharak Amanzadeh, Karen Sokal-Gutierrez, Judith C Barker

**Affiliations:** Office of Dental Health, Alameda County Department of Public Health, Oakland, CA USA; Department of Preventive and Restorative Dental Sciences, University of California San Francisco, School of Dentistry, San Francisco, CA USA; UC Berkeley-UCSF Joint Medical Program, University of California Berkeley, School of Public Health, Berkeley, CA USA; Department of Anthropology, History & Social Medicine, San Francisco, CA USA; Center to Address Children’s Oral Health Disparities, University of California San Francisco, San Francisco, CA USA

**Keywords:** Global health, Oral health, Advertisements, Junk food, Beverages, Snacks, Public health, Children’s health, Nutrition transition, El Salvador

## Abstract

**Background:**

Globalization and increased marketing of non-nutritious foods and beverages are driving a nutrition transition in developing countries, adversely affecting the health of vulnerable populations. This is a visual interpretive study of food, snack, and beverage advertisements (ads) in rural and urban El Salvador to discern the strategies and messages used to promote consumption of highly processed, commercialized products.

**Methods:**

Digital photographs of billboard and wall advertisements recorded a convenience sample of 100 advertisements, including 53 from rural areas and 47 from urban areas in El Salvador. Advertisements were coded for location, type of product, visual details, placement and context. Qualitative methods were used to identify common themes used to appeal to consumers.

**Results:**

Advertisements depicted “modern” fast foods, processed snacks and sugary beverages. Overall, the most prominent themes were: Cheap Price, Fast, Large Size, and Modern. Other themes used frequently in combination with these were Refreshment, Sports/Nationalism, Sex and Gender Roles, Fun/Happy Feelings, Family, Friendship and Community, and Health. In rural areas, beverage and snack food ads with the themes of cheap price, fast, and large size tended to predominate; in urban areas, ads for fast food restaurants and the theme of modernity tended to be more prominent.

**Conclusions:**

The advertisements represented a pervasive bombardment of the public with both explicit and subliminal messages to increase consumerism and shift dietary patterns to processed foods and beverages that are low in micronutrients and high in carbohydrates, sugar, fat and salt—dietary changes that are increasing rates of child and adult diseases including tooth decay, obesity, cardiovascular disease and cancer. Global food and beverage industries must be held accountable for the adverse public health effects of their products, especially in low-middle income countries where there are fewer resources to prevent and treat the health consequences. In addition, public health and governmental authorities should learn from the advertising strategies to promote social marketing of public health messages, and enact and enforce regulations to limit the advertisement and sale of unhealthy products, particularly for children in and around schools. This will create healthier social norms and environments for the entire population.

**Electronic supplementary material:**

The online version of this article (doi:10.1186/s12889-015-1836-9) contains supplementary material, which is available to authorized users.

## Background

Over the past several decades, globalization has led to a dramatic increase in the marketing and consumption of carbohydrate-and fat-dense, low-micronutrient processed foods and beverages, particularly in developing countries [1, 2]. This global “nutrition transition” from the traditional to “modern” diet has been associated with a significant increase in dental caries, obesity, type II diabetes, cardiovascular diseases and cancer, described as “non-communicable diseases” (NCDs), in children and adults [3, 4]. Food and beverage advertisements (ads) have been found to play a significant role in promoting the nutrition transition, contributing to the global pandemic of NCDs [5]. In response, the World Health Organization and many national and local jurisdictions have developed guidelines and regulations addressing the advertising of non-nutritious food and beverages to children [6, 7]. However, implementation of the guidelines has been challenging, especially for low-and middle-income countries that lack the resources to monitor marketing activities or care for their population’s burgeoning chronic health problems.

Advertisements use various techniques to appeal to different populations, adapting to the cultural and social contexts of the target audience [8]. Specific strategies are used to appeal to children, for example. Also, different strategies may be used in low-or middle-income country compared to a high-income country, in urban vs. rural settings, and in different regions and cultures. Ads often target basic human emotions and desires to feel successful, loved and happy—with the message is that consuming a product can be a simple way to fulfill one’s desires [9, 10]. However, this manipulation of information and desires can have serious adverse health consequences.

There is limited literature on food advertisements in developing countries [11, 12]. Some cross-cultural studies have used content analysis to study advertising appeals in different countries, most of them of developed countries [13–19]; some of these studies are of food advertisements specifically [13, 14]. Cheong and colleagues identified cultural dimensions of appeals, including individualism, power and long-term orientation. Advertising appeals within these dimensions included: independence, distinctiveness, family, community, popularity, status, and, health and nutrition [11]. Elliot used content analysis to assess supermarket foods targeted at children, identifying use of “fun” graphics such as cartoon characters, as well as healthy nutrition claims [13]. Aronovsky and Furnham used content analysis to study gender stereotyping in televised food advertisements [20], while Parkin’s *Food for Love* analyzed advertisements for gender-specific messages to women [21]. There remains a need, however, for further study of food and beverage advertisements in the context of developing countries, and rural vs. urban settings. We report here on one such study that begins exploration of these topics in El Salvador in Central America.

El Salvador is a lower/middle-income country, with substantial wealth and health disparities. Nationally, 40 % of the households live in poverty, including 50 % of households in rural areas [22]. Moreover, El Salvador has persistently high rates of child malnutrition— among children under age 5, 19 % nationally and 36 % in rural areas have chronic malnutrition [23]. Food insecurity is frequently exacerbated by environmental disasters such as earthquakes, hurricanes, droughts and floods [24]. In addition, over recent decades, El Salvador has experienced a dramatic rise in obesity, which has been attributed to rapid urbanization and dietary change [25]. Currently, 61 % of adults are overweight, including 26 % who are obese [25]. In children, there was a 50 % increase in obesity from 4 % to 6 % between years 1993 to 2008, with higher rates in urban and higher-income populations [22]. It is predicted that this “double burden of malnutrition” could seriously threaten the health care system’s ability to care for the population’s chronic diseases, and limit the country’s economic productivity. Thus, there is an urgent need for interventions to reverse the unhealthy nutrition trends [25].

The interpretive study reported here is an initial exploration of the ways through which food and beverage advertisements in rural and urban areas in El Salvador interface with the “nutrition transition”.

## Methods

### Study design

This is a visual interpretive study of a convenience (non-random) sample of food, snack and beverage advertisements in a rural and urban region of El Salvador. This study was embedded in a larger study on children’s nutrition and oral health in the Santa Ana region of El Salvador. in collaboration with a local organization, ASAPROSAR. ^a^ This interpretive study aims to explore how highly processed, commercialized foods and beverages are being promoted, the methods and themes used by the advertisements, and how these differ between rural and urban areas.

### Data collection

Photographic data and field notes were collected by one photographer during a 1-week period in July 2010, from six rural villages in the Santa Ana region, the roads connecting these villages to the city of Santa Ana (the second largest city in El Salvador), and the principal road from Santa Ana to the capital city San Salvador. A digital camera was used to photograph billboard and wall advertisements of food, beverages (including beer) and snack products. Additional photographs from both rural and urban areas were taken. These extra photographs were of ads in close proximity to the advertisements in the formal sample, and helped provide contextual information about the how the food and beverage ads were visually displayed. The goal was to capture a full range of the various types of food and beverage advertisements encountered. In rural areas, photographs were taken while walking along small (secondary) roads; in urban areas, photographs were taken from a car travelling on principal (main) roads. This difference reflects the usual ways of commuting in these areas, and the ways people are exposed to the advertisements.

Consents and Ethics policies are not applicable to this study, as human subjects were not involved.

### Data analysis

When the same advertisement occurred in several places and was recorded several times, duplicate photographs were eliminated so that the final dataset comprised single representations of specific advertisements. Through qualitative analysis of the visual content of the photographic images captured, we identified patterns and explored the themes, topics and symbols revealed in the advertising [26, 27]. Similar to Parkin [20], our study identifies the broad messages that food advertisements convey. Our analysis applied an interpretive approach similar to that used by Cheong and colleagues [10], by Elliot [12] for advertising appeals, and by Aronovsky and Furnham [19] for the target population. As Rose [28] suggests, the meanings of an image are made at three different sites: “the site(s) of the production of an image, the site of the image itself and the site(s) where it is seen by various audiences”. She also suggests that “each of these sites also have three different aspects or modalities that contribute to a critical understanding of image: technological, compositional, and social”. Our analysis approach focused on the sites of the image and the audience as well as compositional and social modalities as they related to the advertisements.

Each advertisement was categorized for location (as rural or urban; and whether it was placed on a wall, billboard or other object) and type of product (food, snack or beverage). Each was analyzed and coded for its visual details such as color, size, design and content, for placement and context, for the meanings of the words used, for what attracted the most attention in a single advertisement, and for the relationships observed among adjacent advertisements. Then the inferential meaning and the main theme of each advertisement were identified. Ads were initially coded for their strongest, most prominent themes. Further rounds of thematic analysis were then used to identify up to two more minor themes, and the interconnections among all themes. Coding ceased when no new themes or connections could be identified across the advertisements.

## Results and Discussion

In this section, we first present a general description of the advertisements, their location, and the main themes discerned. For the purposes of this analysis, we focus on just two locations–rural villages and their nearby small or secondary roads, and urban cities and their nearby principal roads. Intermediate or peri-urban locations are not addressed in this study. Each theme is presented and, where pertinent, a photograph illustrating the theme is provided. In some instances, brief and directly relevant comments are made about a specific theme with a more extensive commentary appearing later. As shown below, themes were manifest differently in rural vs. urban ads, in terms of objects upon which the ads were placed, type of food or beverage advertised, the choice and frequency of appeals, the approaches used and the meanings conveyed.

### General description of advertisements

We analyzed 100 different advertisements for fast food, snack food and beverages, including 53 from rural areas and 47 from urban areas (Table 1). Rural ads were predominantly for beverages, followed by snacks. In rural areas, fast food ads were not seen, likely due to the lack of such establishments in rural settings. In contrast, in urban areas where fast food restaurants were located, urban ads consisted mostly of fast food ads for chain or franchise quick-service restaurants and independently-owned outlets, followed by snack and beverage ads. An additional 21 photographs, from both rural and urban locations, were examined to further discern not the thematic content but rather the visual context of our sample. These extra photographs/ads tended to depict non-food images -- such as tobacco products, mobile telephones and medicines–placed in close proximity to the food or beverage ads of interest. This broader visual context bolstered our understanding of the context of the ads and the recognition of one major theme–namely, modern–in the food and beverage ads of interest.

**Table 1:** General description of convenience sample of food, snack and beverage advertisements (n = 100 photographs of ads)

Geographic setting	Type of product advertised		
	Fast food	Snacks	Beverages
Rural (n = 53)	0	31	40
Urban (n = 47)	31	9	7

Note: Duplicate photographs were removed, so each image represents a separate, independent advertisement

### Context of rural and urban ads

In the rural villages, advertisements were posted primarily on the walls of small local stores where local residents purchased household necessities and chatted with neighbors, and children bought snacks. Ads were also mounted on trees close to the shop, but advertisements generally were on the store wall, clustered around the main window, appearing like an octopus, spreading its tentacles across the wall. There was obvious motivation for ad placement to attract attention–to be more central, approximately at eye level, to have a bigger display, to have more eye-catching photographs or graphics, or to advertise a cheaper price than competitors. The villagers, mostly farm-workers and their children were the main audience for the ads. (Additional file 1).

Since many people drove or took buses within and between the main city of San Salvador and smaller towns such as Santa Ana, the ads were generally placed along the route of the main rural-urban roads—on high roadside billboards between cities, and on lower billboards in the towns where the traffic is slower. Some ads were repeated on a succession of billboards. Ads were especially prominent around major urban intersections, where they could be seen above the traffic and viewed for a longer time while waiting at a stoplight. (Additional file 2).

### Advertising themes for food, snacks and beverages

Eight prominent themes emerged from analyzing the appeals of the advertisements (Table 2). The importance and order of the themes was determined based on the overall frequency of appearance of a specific theme in the total sample of ads. The most frequent themes, assumed to be the most important, were: Cheap Price, Fast, Large and Modern. Other themes were commonly used in combination with these primary or frequent themes: Refreshment, Sports/Nationalism, Sex and gender roles, Fun/Happy feelings, Family, Friendship and Community, and Health.

**Table 2:** Advertising themes for foods, snacks and beverages, by frequency (%) for rural vs. urban distribution of ads (n = 100 advertisements)

Advertising themes	Frequency (%) across all themes	Distribution of theme, by rural versus urban area
1. Cheap price, large, fast	39 %	Both, more in rural
2. Modern	16 %	Both, more in urban
3. Refreshment	10 %	Both
4. Sports/Nationalism	9 %	Both, more in urban
5. Sex and gender roles	7 %	Both, more in rural
6. Fun/Happy feelings	7 %	Both
7. Family, Friendship and community	7 %	Both
8. Health	5 %	Both, more in rural

Note: These percentages represent the proportion in this study’s non-random sample, not the distribution of these items in the total population of relevant advertisements in the locations studied

#### Cheap price, large and fast

Numbers had a significant presence in the ads, communicating two things: money and time, or “cheap price” and “fast”. Numbers written in a large font against a contrasting background grabbed one’s attention. In a fast food advertisement for a hamburger (See Additional file 3), half of the billboard was a picture of a hamburger; the other half was a picture of a bottle of Pepsi**®** and French fries, surrounded with a few words. These words were in large fonts and in red against a white background, emphasizing the price and size. From a distance, one saw the hamburger, the price: “$2.49”, and a statement: “Ahorro Combo”, or Savings Combo. This is a simple and effective way to appeal to the low-literacy population traveling from rural areas to the city to sell their products and buy necessities. In addition, the association of the cheap price with the disproportionately large picture of a hamburger exaggerates the good value for the money.

#### Modern

Modernity was used as an attractive appeal, especially but not only for the urban population. A roadside billboard for fast food, near the capital city, showed a hamburger on a plate surrounded by images of famous European buildings. The food was advertised as “The European Sandwich” (See Additional file 4). The ad appealed to the customer’s aspirations for a modern and sophisticated life style (such as being affluent and having social status) associated with Europe. With the ad’s unnatural proportions—the buildings dramatically scaled down and the hamburger dramatically scaled up—the image shouted out the importance of the hamburger in the context of the modern world.

#### Refreshment

The refreshing quality of beverages was highlighted in many rural and urban ads. This message was visually transmitted through the color or foaminess of the drink, a glass that was frosted or had beads of condensation, or bubbles in the background. It was also conveyed through words that highlighted “thirst” or “refresh”. The association between the aching of thirst and the joy of slaking one’s thirst reminds the viewer of the continual need for hydration in the warm Salvadoran weather, especially when doing physical labor or engaged in sports (Additional file 5).

#### Sports and nationalism

The Refreshment appeal was strengthened by combination with sports images and integration with sports events (Additional file 5). The image showed national sports team members with strong-looking faces and bodies painted with the national flag. The beverage logo was seen in four places in the ad, against a blue, bubbly background. On top, it announced, “Refresh your passion”, which implied passion for sports, national pride, love or sex. On the bottom, it stated, “Refresh your world”, communicating a message that this is your world and your identity, and this powerful feeling comes from consuming this beverage.

#### Sex and gender roles

Sex and gender roles were used primarily in ads for soda beverages. In some ads, the inclusion of or nearby placement of ads for beer provided a context that reinforced these sex and gender roles. Most cultures have explicit or implicit generalized gender roles, and advertisements can reinforce or contradict these gender roles [29].

Sex-based themes and gender roles have been widely used to promote products in advertisements throughout the world [30–34]. In rural Salvadoran advertisements, sex appeal was conveyed with an unusual rawness. A soda ad on the wall of a small rural store (Photo 6) showed a young woman, who is light-skinned, with long black hair, wearing a white bikini, on the beach. The white bikini blended with her skin, making the model appear naked at first glance, drawing the viewer into looking again. Above her abdomen and legs were the images of three large soda bottles with their prices. The ad asked the viewer to “enjoy”, and “share it with ice”. The combination of the image and the concepts of enjoyment and sharing can stimulate feelings of desire. In rural areas where women dress very conservatively, this advertisement appeared almost pornographic, attracting men’s attention. As apparent in the photo, the ad was subject to graffiti, confirming that it drew attention from viewers.

In other ads, however, the audience seemed to be women themselves, offering them a glimpse of a modern identity. The ad depicted in Additional file 6 showed a close-up of the face and upper body of a young Latina-looking woman with long black hair, eyes closed, head up, drinking soda from the bottle, similar to the male athletes in other ads. This ad presented an image to which young rural women could aspire–looking modern and sexy, and boldly swigging soda.

#### Fun and happy feelings

Snack foods were generally portrayed as fun to eat (Additional file 7). These ads used bright colors and cartoon figures, which attracted attention, especially from children. An ad for ice cream showed the variety of flavors available, in different shapes and colors. In another ad, emoticon cartoon faces–happy, angry, surprised, and cool–surrounded the image of a bag of snacks, a rabbit introduced chocolate milk, and a soccer ball-shaped bag connected the “yummies” with playing soccer (Additional file 8). These ads all used vibrant colors: pink, purple, light blue, light green, and bright red.

Beverage advertisements also used “fun and games” as an appeal, but not as widely as for the snack category. When used, the beverage “fun and games” appeal mainly targeted children and young adults. Some ads offered games such as collecting bottle caps, with a reward for finding specific ones. Visually, these ads also used bright colors such as yellow and red as well as cartoon images, and combined the message with sports imagery and specific events, such as the soccer World Cup (Additional file 8).

#### Family, friendship and community

The concept of Family, Friendship and Community was an appeal emphasized in some advertisements. A beverage ad showed a well-dressed, presumably middle-class family sitting at a table having beer with their dinner (Additional file 9). The ad said, “Escógela a tu medida”, or ‘choose it according to your needs’. The ad hinted at gender and family roles in a modern setting, and the power of beer to facilitate social and family relationships. The ad also used the element of affordability, which could make it more appealing in a rural setting.

In a restaurant, shiny round red tables with the red and white Coca Cola**®** logo on them mixed functionality and advertising (Additional file 10). The Coca Cola**®** tables facilitated social gatherings by providing a place for families and friends to enjoy a meal together, while prominently advertising this product.

#### Health

Appeals to better health were not common among the advertisements in our study, in contrast to high-income countries where ads commonly tout the nutritious qualities of food and beverages to attract consumers [35]. In our sample though, appeals to health were tied to modernity–for example, one fruit drink had the word “California” at the bottom on it, another advertised having vitamin C, which was said to be “nuevo” or new.

In addition, health products were advertised on the walls of rural stores, lending legitimacy to the food and beverage ads through visual and placement strategies. As seen in Additional file 11, ads promoting a variety of snacks and beverages had legitimacy conferred on them because of their placement near a health product. An “Alka-Seltzer**®**” advertisement, posted higher than any other ad, and with large fonts and solid colors, dominated but legitimized the rest of the ads.

### Communicative strategies

Advertisements used different communicative strategies or techniques to maintain their visibility or reinforce the message for their audience. Table 3 outlines the various communicative strategies observed in our sample.

**Table 3:** Communicative strategies of advertisements for food, snacks and beverages *(n = 100 advertisements)*

Main communication strategy used in each advertisement	Percent of all strategies
Combination	35 %
Repetition/Resonance	20 %
Placement/Visibility	18 %
Personification	15 %
Redefining food	12 %

Note: These percentages represent the proportion in this study’s non-random sample, not the distribution of these items in the total population of relevant advertisements in the locations studied

#### Combination

Combinations of different appeals tapped into multiple emotions and reinforced key messages. Combining appeals could provide an engaging story line with a single conclusion: the urge to buy and consume the product. For example, ads that combined Refreshment and Sports were reminders of one’s thirst and need for hydration, and asserted that drinking this beverage would alleviate one’s thirst and express pride for the national sports team (Additional file 5).

#### Repetition/Resonance

Repetition of advertisements in different places throughout the environment conveyed a sense of omnipresence and normalcy, demonstrating that the product was an integral part of daily life. For example, beverage advertisements for soda, sports drinks and alcohol were seen on billboards and store walls throughout the community, suggesting that these were the beverages one should drink every day, in contrast to water or milk, which were rarely seen in ads.

“Resonance”, as defined by McQuarrie [36], “occurs when there is a repetition of elements within an ad, and when this redundancy is such that an exchange, condensation or multiplication of meaning occurs”. Simple repetition is not sufficient to create resonance–repetitive elements must also echo one another, modify or re-contextualize the meanings that each would have had alone [36]. The appearance of multiple soda ads in different forms and contexts supported a resonant effect. For example, on a rural store wall, several soda advertisements occupied the most visible spots in the middle of the wall; and one soda ad had a logo that was repeated in each of the four corners of the ad.

#### Placement/Visibility

Ads aimed to integrate their message into the consumers’ daily lives. Placement of ads in prominent places throughout the community reinforced this integration and associated the product with specific community activities. For example, in both rural and urban settings, snack food and soda companies advertised on umbrellas and trucks at outdoor markets, community centers and events. The ubiquitous presence of these ads throughout the community reinforced the message that these products were integral to daily life and community celebrations, and that rural and urban communities were united by the snacking culture.

#### Personification

Personification is the use of familiar people or cartoon characters to help make a product seem familiar and desirable [37]. Seeing a familiar face, such as a famous person endorsing a product, can establish the value of the product and make one more likely to want that product. In addition, the characteristics of the person can be transferred to the product; for example, endorsement by an athlete implies that the product is healthy [38]. Cartoon characters can particularly appeal to children, invoking a cute, funny or fun association with the product [39]. In our sample of ads, personification was seen clearly in the cartoon characters used for advertisements for snacks, and athletes used in the advertisements for beverages.

#### Redefining food and meals

Ads contribute greatly to the global trend in redefining food and meals. Whereas the traditional Latin American culture valued home-cooked meals consumed together with the entire family over a relaxing several-hour mid-day “siesta” break [40], the ads promoted the modern lifestyle with fast food meals and snacking on-the-run. This nutrition transition to fast food and snack food was seen most prominently in the urban areas, but had begun to extend to the rural areas as well.

Some ads promoted a bridge from the traditional to the modern diet through a combination of modern and traditional foods, and Spanish and English words. One fast food ad showed a “Big Burrito” combo with French fries and soda, naming it the “Nuevo (new) Meal”. The burrito, although appearing to be more traditional Latin American food, is in fact an American version of Mexican food. Combining it with other elements of modern fast food–French fries and soda–produced a modern combination with traces of authenticity [41].

Fast food establishments also redefined their food offerings to appeal to the consumers’ appetites at any time of the day. For example, pizza chains sold breakfast items, encouraging dining-out for breakfast, not previously part of the local culture. The dining experience had also been redefined by associating it with other activities. For example, several fast food establishments added play structures for children, making the restaurant a fun destination for families.

### Food, snack and beverage advertising in El Salvador: increasing consumerism and shifting dietary patterns

This study explored food, snack and beverage advertising in El Salvador as a means of communication, and as a chronicle of the processes of globalization, urbanization and dietary change. This study had some limitations. It used a small convenience (non-random) selection of accessible food and beverage advertisements in both rural and urban environments in a particular region in El Salvador. As such, it is not representative of all such advertisements in that country, nor of their distribution across various areas. Nevertheless, this interpretive study makes a contribution to the literature through its identification of common advertising themes, provision of brief illustrative examples and explanations for each theme. To our knowledge, it is one of very few studies that have examined the visual communicative strategies used in food and beverage advertisements in a low-to middle-resource country.

Overall, the ads represented a pervasive bombardment of the public with both explicit and subliminal messages. Explicit messages tended to be factual; for example, showing the cost of a particular size bottle of soda, as in Additional file 12. Subliminal messages were more subtle in their appeal, such as the suggestion that women can demonstrate or adopt a modern identity if they consume soda in particular fashion, as depicted in Additional file 6. Both types of message are intended to increase consumerism and shift dietary patterns to processed foods and beverages that are low in micronutrients and high in carbohydrates, sugar, fat and salt.

There were some similarities and differences in both the products and kind of ads in rural and urban areas. While rural ads tended to promote more affordable products such as soda and snack foods, urban ads predominantly promoted fast food restaurants selling foods such as European sandwiches and pizza. Fast food advertisements consistently used the themes of cheap price, large size, and modernity, often combining themes for greater appeal. Beverage advertisements commonly used appeals of refreshment, cheap price, sports, and sex. Snack ads generally used themes of fun, modernity, and variety, with ads targeting children using bright colors and cartoon characters.

The communication strategies found in these ads aimed to affect the emotional response of consumers. Tapping into emotions can be direct or subtle, as seen in slogans such as “Despierta la alegría” or “Awaken joy”, and “Disfrútalo de nuevo” or “Enjoy it again”. Multiple emotions were evoked simultaneously, further heightening the impact and appeal of the advertisement. Strategies seemed to differ slightly from rural to urban areas, being adapted by the advertisers to the environment, to appeal to the needs and desires of local consumers. While advertisements presented the facts regarding the pricing of products, the creative visual presentation and messaging appealed to the consumers’ emotional motivation to buy the cheaper or bigger product, in order to get the best deal or “save money”, which is particularly important for low-income families. In this lower/middle-income country, with an average income of less than $10/day [42] and even less in the poorer, rural areas, it is noteworthy that families are willing to spend 5 %–10 % of their daily income on a single soda or snack.

Advertising is a powerful tool for conveying messages to a broad audience—a mirror for cultural communication [43]. However, “the mirror is distorted… [it] serves the seller’s interest [43]”. Advertising relies on persuasive and symbolic images to sell products by “associating them with certain socially desirable qualities, but they sell, as well, a world view, a life-style and a value system congruent with the imperatives of consumer capitalism [44–46]”. Some argue that both advertisers and people seeking information and interpreting the advertisements together create this meaning, and thus the production of advertisements is a joint effort [47]. However, in the context of a developing country, it is mostly a one-way transmission of cultural values from advertisers and companies, many of which are multinational [48] promoting consumerism at the expense of traditional culture and health [49, 50].

In El Salvador, with its warm climate, the need for hydration is critical, especially for people employed in physical labor. In many rural areas, however, there were insufficient sources of affordable clean water such as the tap, wells or bottled water. While ads for soda were ubiquitous in both rural and urban settings, we found only one ad for water—a small blue-and-white, rather dull picture of a bottle, priced at almost twice the cost of a can of soda. This disparity in price between water and soda had been repeatedly noted throughout the field research period of this study. In addition, the sugar and caffeine in the soda could provide immediate sources of energy, and suppress appetite for families who frequently lack sufficient food. The marketing strategy of large, cheap and accessible soda takes advantage not just of families’ needs for hydration but also implies a sensible deployment of their limited monetary resources.

Furthermore, images of athletes and sports were commonly used to convey a message of healthiness to sell beverages, including soda and alcohol. The images of athletes portrayed youthfulness, strength, heroism, popularity, and health. The ads reinforced the refreshing quality of the drink and combined an emotional appeal to the youthful passion for sports along with admiration of prominent athletic figures. Globally, the sports industry has benefited from its advertising partnerships with soda, alcohol and tobacco companies, belying the adverse health consequences of these products [51, 52]. World Cup soccer themes appeared widely in El Salvador, with images of the victorious players, a soccer ball, friends and family watching a game together, or as a game for children to collect bottle tops for different teams. The FIFA logo confirms the association between the beverage and sports industry, and between drinking particular products and enjoying the games. The ads implied that even long after the game ended, drinking this beverage could bring back the memories and enjoyment. This association between unhealthy food, drink and snacks and national sports figures is similar to the strategies that the tobacco industry has used in its advertising [53]. In both cases, sports have been used to promote products that have increased the burden of chronic diseases in low and middle-income countries.

In addition, El Salvador has longstanding ties to the United States. The country received financial and military support from the US during its civil war from the 1970s to 1992. It adopted the US dollar in 2001, ceding control over monetary policy, and ratified the Central American Free Trade Agreement (CAFTA) in 2006, expanding imports and exports [54]. US-based or multi-national food and beverage manufacturers, distributors or franchises are among the commercial enterprises that benefit from this policy. Trade with El Salvador because of CAFTA permits not just the easier exchange of money, products and people but also the transmission of “modern” ideas and behaviors, including dietary practices and preferences. A study by Offer and colleagues stated that countries with “market-liberal welfare regimes tend to have the highest prevalence of obesity”, influenced by the prevalence of fast food, food insecurity and economic inequality [55]. The impact of CAFTA on employment, production and poverty in El Salvador could help provide another explanation for the lower price of soda than bottled water, a phenomenon observed in the field [56]. Additionally, as one-third of Salvadoran households have family members living in the US, and financial remittances constitute 16 % of gross domestic product [57], US dietary values can easily be transmitted to El Salvador to become pervasive influences, capable of penetrating deeply beyond the urban centers.

Food, drink and snack advertisements contribute to changing the culture around food and beverages by promoting a “modern” diet that is high in fats and sugars, and low in whole grains and fiber—with the implicit message that the modern diet is high-status, tasty and desirable, while the traditional diet is outdated, low-status and undesirable [58]. In addition to the loss of the native cultural traditions, this modern diet (including foods and beverages) dramatically increases the risk for obesity and other “non-communicable diseases” such as type II diabetes, cardiovascular disease and cancer [59, 60]. Another less-discussed consequence of the modern diet is dental caries or tooth decay, which has become the most prevalent chronic disease worldwide [61, 62]. Due to the dramatic increase in bottle-feeding and sugar/carbohydrate-dense snack foods and beverages for children, tooth decay commonly begins within the first 2 years of life, and affects 60–95 % of children by age 6. The consequences of early childhood caries can be severe, including mouth pain, and difficulty eating, sleeping and concentrating in school [63]. Unfortunately, in many developing countries access to oral health services is limited, and decayed teeth are usually left untreated or extracted because of pain or discomfort [64].

### Recommendations

Within the field of public health, there is growing awareness of the need for “social marketing” of public health messages, applying successful marketing strategies to promote social good rather than profit [65, 66]. In low-and middle-income countries, there is a need for widespread social marketing to improve nutrition. Social marketing efforts need to utilize strategies such as collecting data on target populations and identifying priority groups; to work with consumers to create tailored, engaging and persuasive messages to promote positive behavior changes; and to continually re-evaluate and tailor the messages [65–67]. Nutrition promotion initiatives should learn from the fast food and beverage advertisers and adopt similar marketing strategies. All types of media (print, television, and electronic) should be employed to address a wide audience. Catchy slogans, famous people and cartoon characters as well as attractive images and bright colors should frame and deliver the messages. Messages should be reinforced by appearing in multiple environments, and should demonstrate clear value for the consumers’ health and well-being (e.g., a healthy smile for children). Social marketing of healthy nutrition must target the general population, school children, vendors and policy-makers to create healthier social norms and environments that make healthy choices the easy choice [65–67].

In recent years, several Latin American nations have recognized the extent of the “nutrition transition” problem, and introduced healthy food laws to try to combat childhood obesity [68]. The implementation of such laws has proven challenging [69]. El Salvador is part of a 2012 Central American Technical Regulation that created rules on advertising claims, prohibiting the promotion of excessive consumption of foods or poor dietary practice; but the regulations lacked clear nutritional criteria to define “unhealthy” foods, limiting enforcement [69, 70]. In 2012, Chile passed a comprehensive law on food labeling and advertising that included defining and posting warning labels on “unhealthy” foods high in calories, sugar, saturated fat and sodium, and decreasing their marketing to children. Industry lobbying, however, led to limits on the foods covered by the new warning labels and permitted toys to be associated with fast food aimed at children [71]. A 2013 law in Peru called for multiple strategies including nutrition warnings on processed foods and beverages, nutrition education in schools, healthy food in school kiosks or cafeterias, and controls on advertising aimed at children and adolescents, but implementation of the law awaits drafting and approval of regulations by a multi-agency commission [72]. Low and middle-income countries, such as El Salvador, should be encouraged to continue introducing and enforcing laws that regulate the marketing of processed, highly commercialized foods and beverages by national and multi-national companies, especially when these products are of low nutritional quality.

## Conclusions

Marketing of “modern” foods and beverages that are high in sugar/carbohydrates and fat, and low in micronutrients, is contributing to the global nutrition transition in low and middle-income countries. While the food and beverage advertisements appeal to peoples’ desires to be modern, successful and happy, and being thrifty through purchase of ‘cheap’ and ‘convenient’ foods and beverages, the adverse consequences are real and can be severe. Outcomes of the shift to the modern diet include additional expenses for food and beverages, loss of cultural practices, and risk for chronic diseases such as tooth decay, obesity, hypertension, heart disease, type II diabetes, and cancer.

There is a need for further interdisciplinary study of the drivers of the nutrition transition in low and middle-income countries, and effective interventions to prevent this unhealthy trend and its adverse health consequences. In an era of globalization, with the development of communication technology, and the expansion of multi-national corporations into “emerging markets”, [73] most developing countries have limited resources to promote healthy diets, regulate and monitor food and beverage advertisements, and provide medical/dental treatment for the chronic diseases resulting from the nutrition transition [74]. Global marketing of food and beverages must be monitored by both global and local agencies, and be held accountable for preventing and treating the adverse health consequences in vulnerable populations.

## Endnote

^a^Asociación Salvadoreña Pro-Salud Rural (Salvadoran Association for Rural Health, ASAPROSAR) is a Salvadoran non-governmental, non-profit organization founded in 1986. ASAPROSAR works with the families in need in El Salvador to improve their quality of life through health care, early childhood programs, youth leadership training, environmental and nutritional education, micro-credit, and community development.

## Additional files

Additional file 1: **Wall of a small shop in a rural setting.**

Additional file 2: **Advertisements in the urban setting of Santa Ana.**

Additional file 3: **Cheap Price and Large Size themes in an urban ad.**

Additional file 4: **Modern theme in ad for fast food.**

Additional file 5: **Refreshment and Sports theme in a billboard frame advertisement.**

Additional file 6: **Coca Cola** **®** **ad, focusing on a woman’s identity.**

Additional file 7: **Fun and Happy Feelings theme manifested in the ads in a rural setting.**

Additional file 8: **Fun and Sports themes in a soda ad in a rural setting, aimed at children: offering the collection of Soccer World Cup team souvenirs.**

Additional file 9: **Family/Friendship themes in a beer ad in a rural setting.**

Additional file 10: **Coca Cola** **®** **tables in a restaurant close to Santa Ana.**

Additional file 11: **Alka Seltzer** **®** **ad on top of the window in a rural setting.**

Additional file 12: **Sex Appeal, combined with Cheap Price theme used in a soda ad in a rural setting.**
