# Supplementary figures and images for: An interpretive study of food, snack and beverage advertisements in rural and urban El Salvador
Source: BMC Public Health. 2015 May 30;15:521. doi: 10.1186/s12889-015-1836-9 (PMC4449567; doi:10.1186/s12889-015-1836-9)

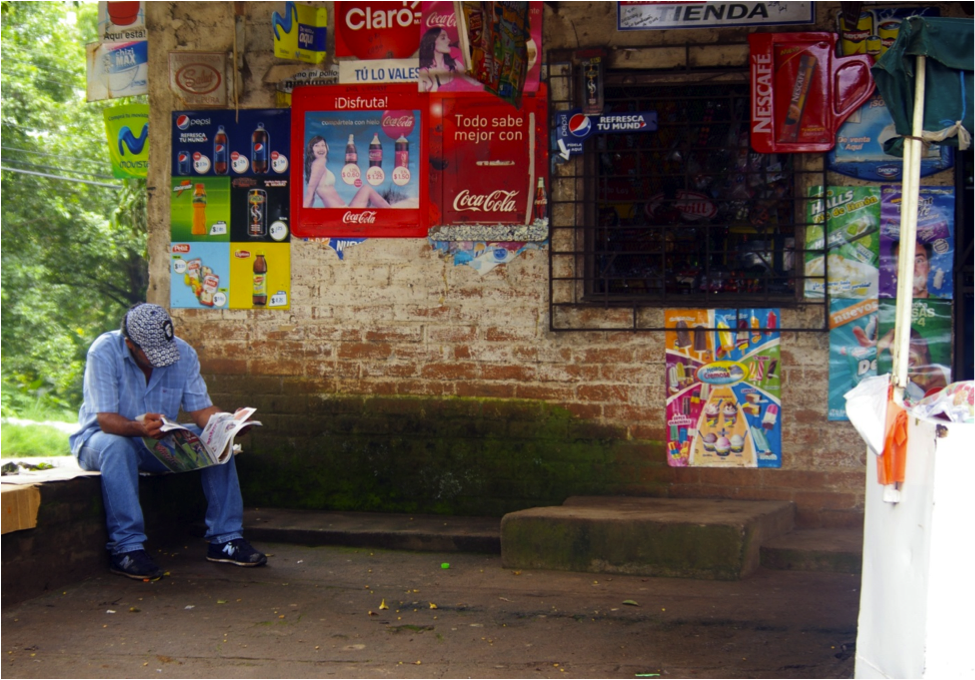

Supplement: Supplementary file 1 — Wall of a small shop in a rural setting. [file 12889_2015_1836_MOESM1_ESM.png]

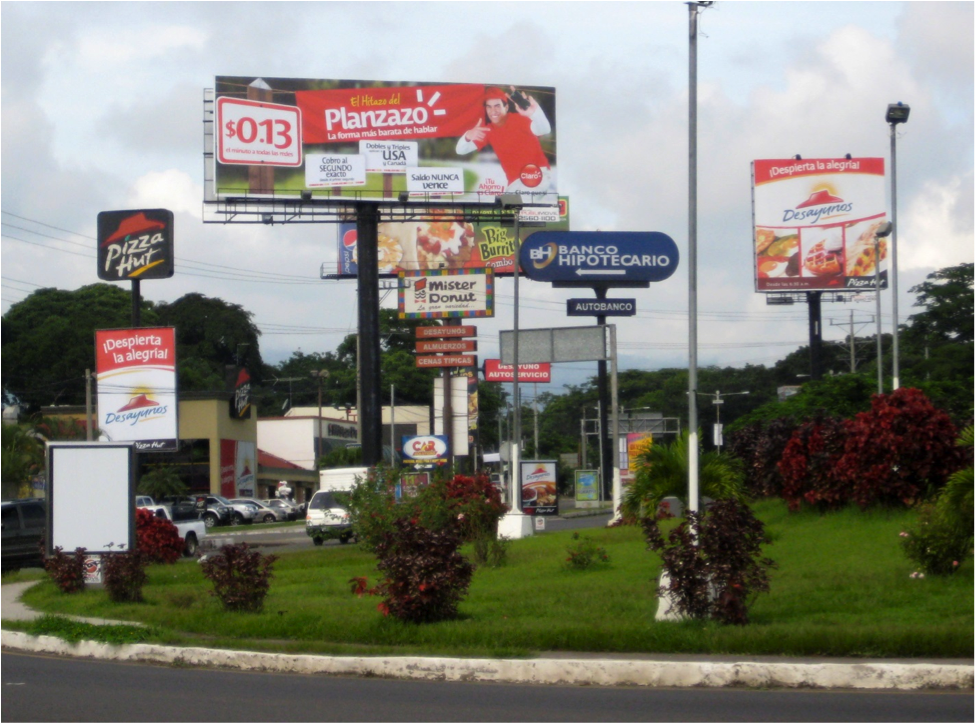

Supplement: Supplementary file 2 — Advertisements in the urban setting of Santa Ana. [file 12889_2015_1836_MOESM2_ESM.png]

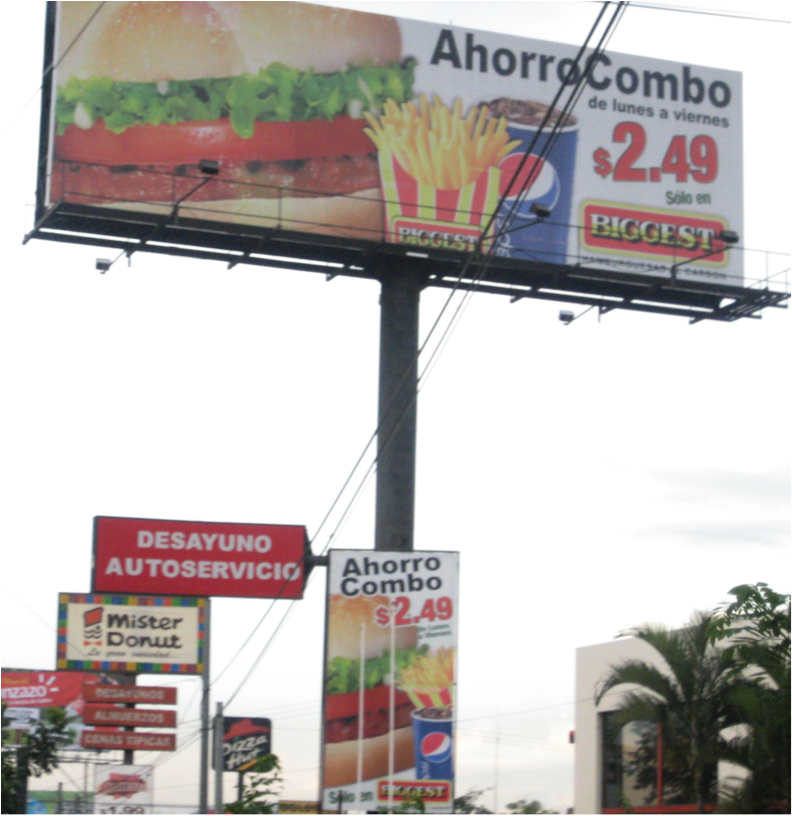

Supplement: Supplementary file 3 — Cheap Price and Large Size themes in an urban ad. [file 12889_2015_1836_MOESM3_ESM.png]

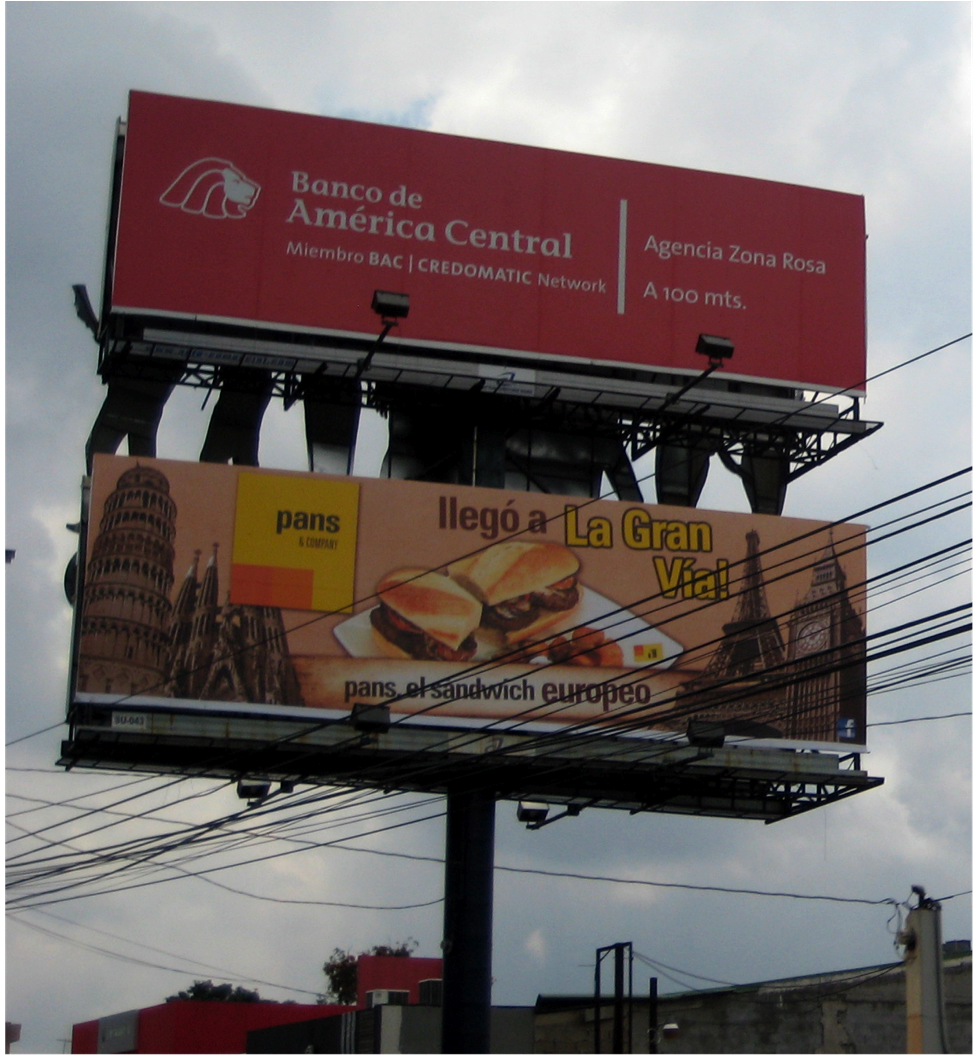

Supplement: Supplementary file 4 — Modern theme in ad for fast food. [file 12889_2015_1836_MOESM4_ESM.png]

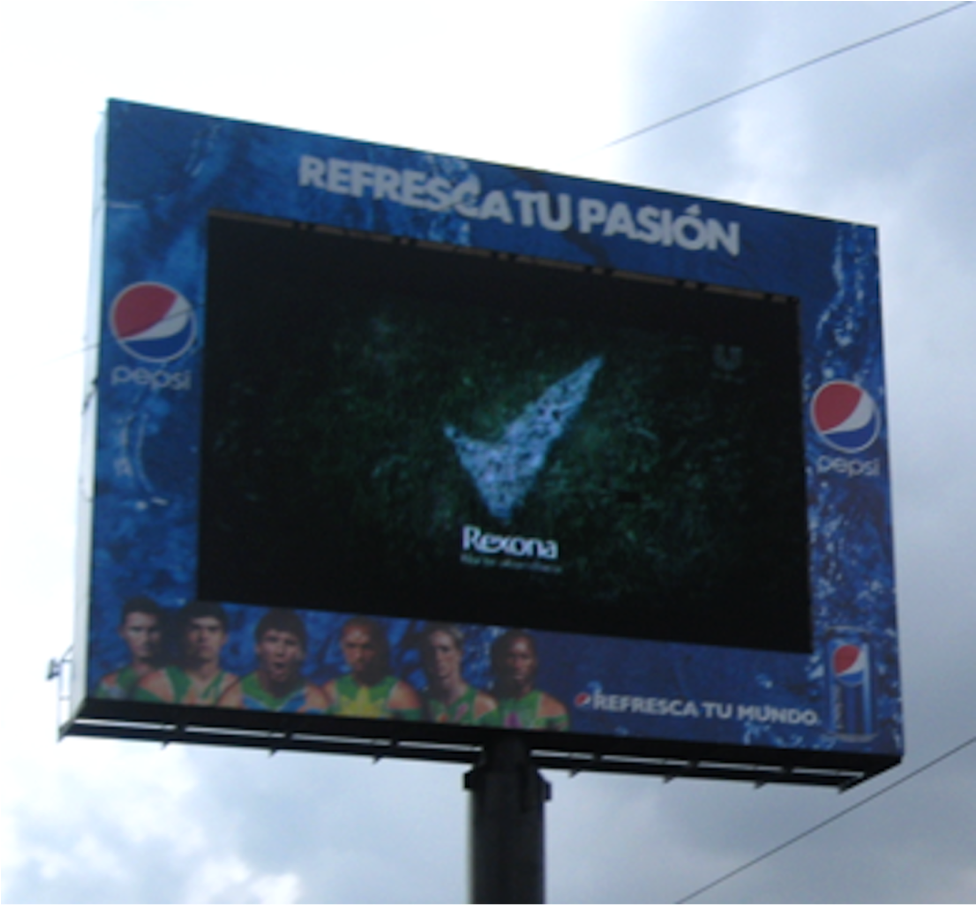

Supplement: Supplementary file 5 — Refreshment and Sports theme in a billboard frame advertisement. [file 12889_2015_1836_MOESM5_ESM.png]

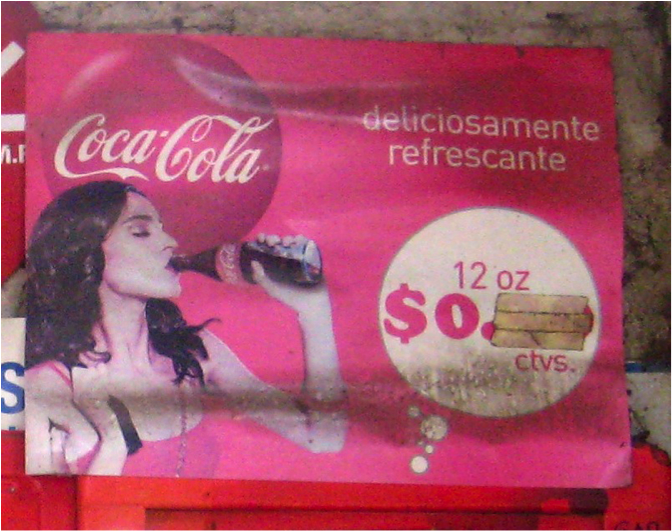

Supplement: Supplementary file 6 — Coca Cola ® ad, focusing on a woman’s identity. [file 12889_2015_1836_MOESM6_ESM.png]

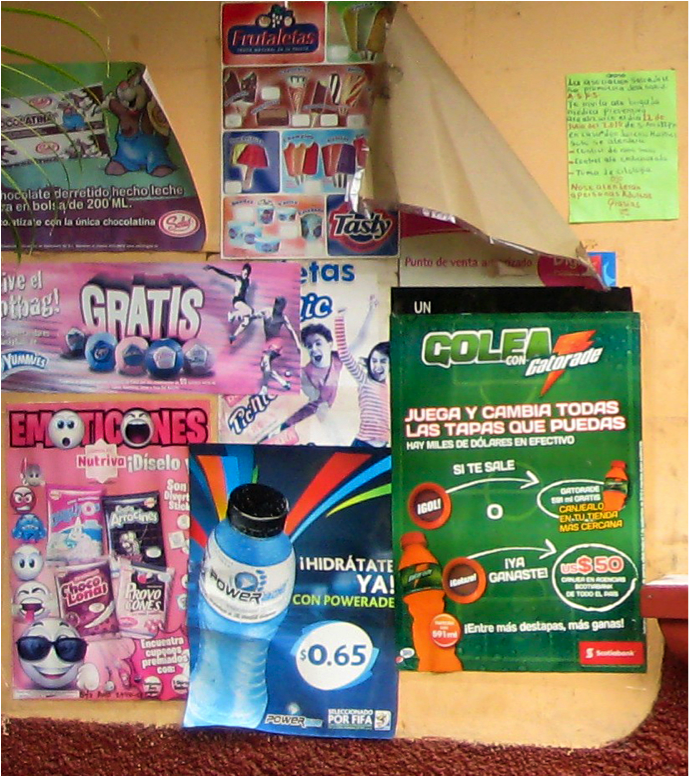

Supplement: Supplementary file 7 — Fun and Happy Feelings theme manifested in the ads in a rural setting. [file 12889_2015_1836_MOESM7_ESM.png]

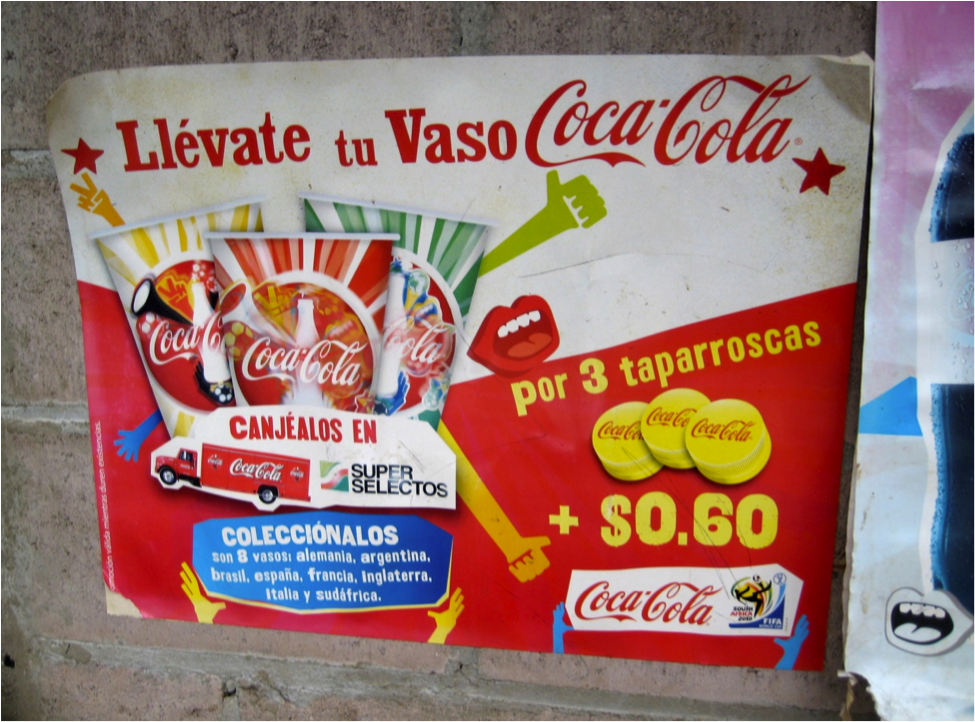

Supplement: Supplementary file 8 — Fun and Sports themes in a soda ad in a rural setting, aimed at children: offering the collection of Soccer World Cup team souvenirs. [file 12889_2015_1836_MOESM8_ESM.png]

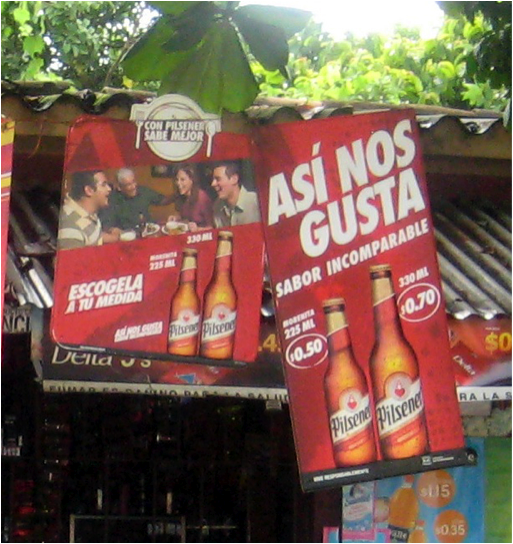

Supplement: Supplementary file 9 — Family/Friendship themes in a beer ad in a rural setting. [file 12889_2015_1836_MOESM9_ESM.png]

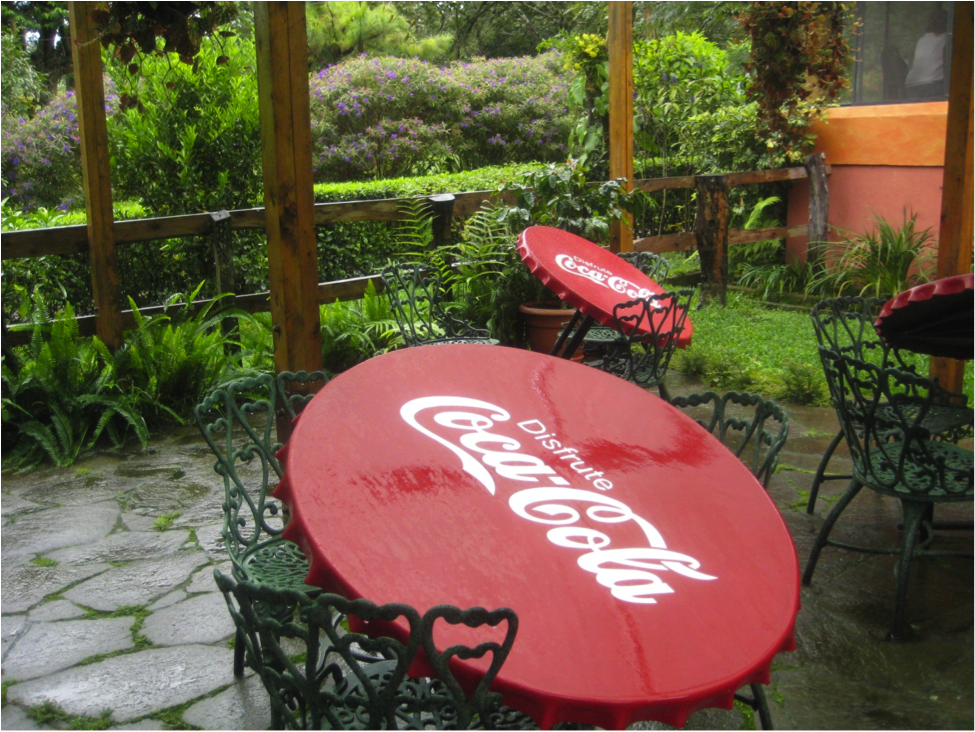

Supplement: Supplementary file 10 — Coca Cola ® tables in a restaurant close to Santa Ana. [file 12889_2015_1836_MOESM10_ESM.png]

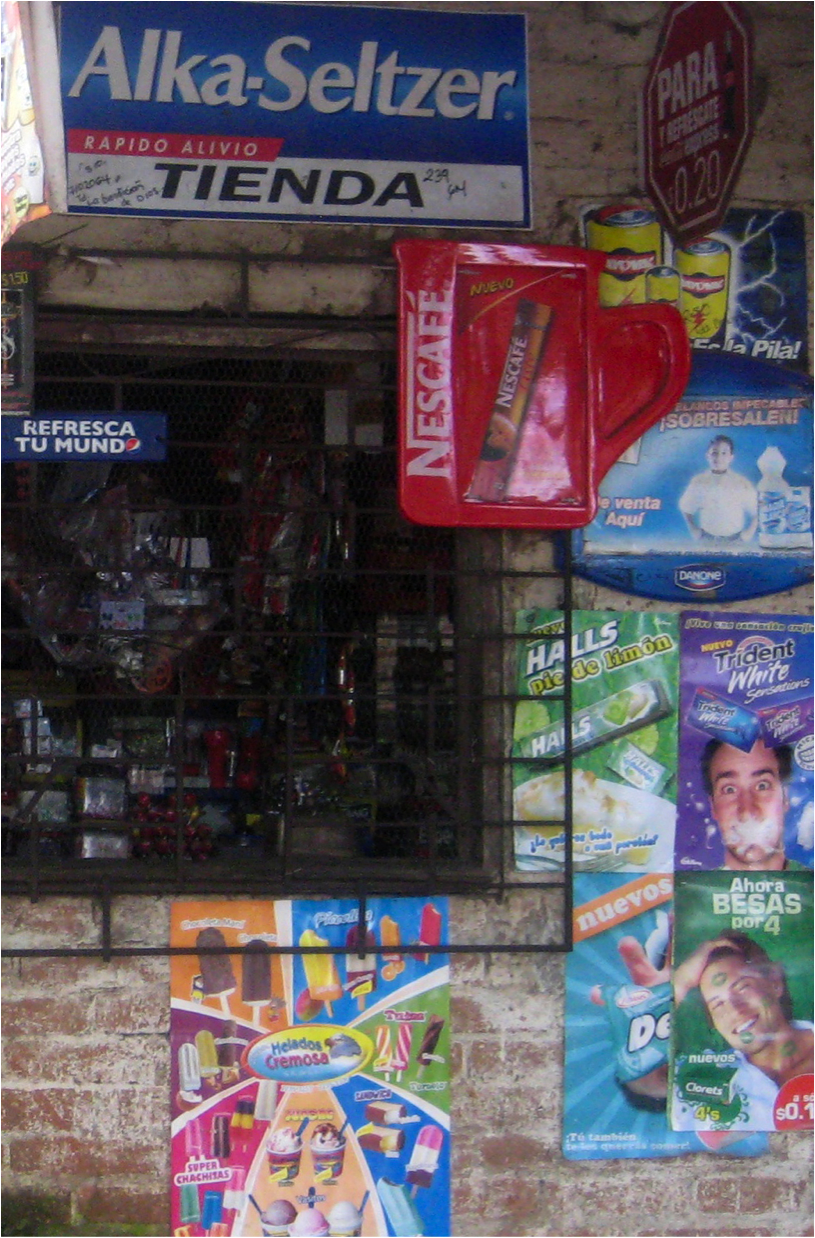

Supplement: Supplementary file 11 — Alka Seltzer ® ad on top of the window in a rural setting. [file 12889_2015_1836_MOESM11_ESM.png]

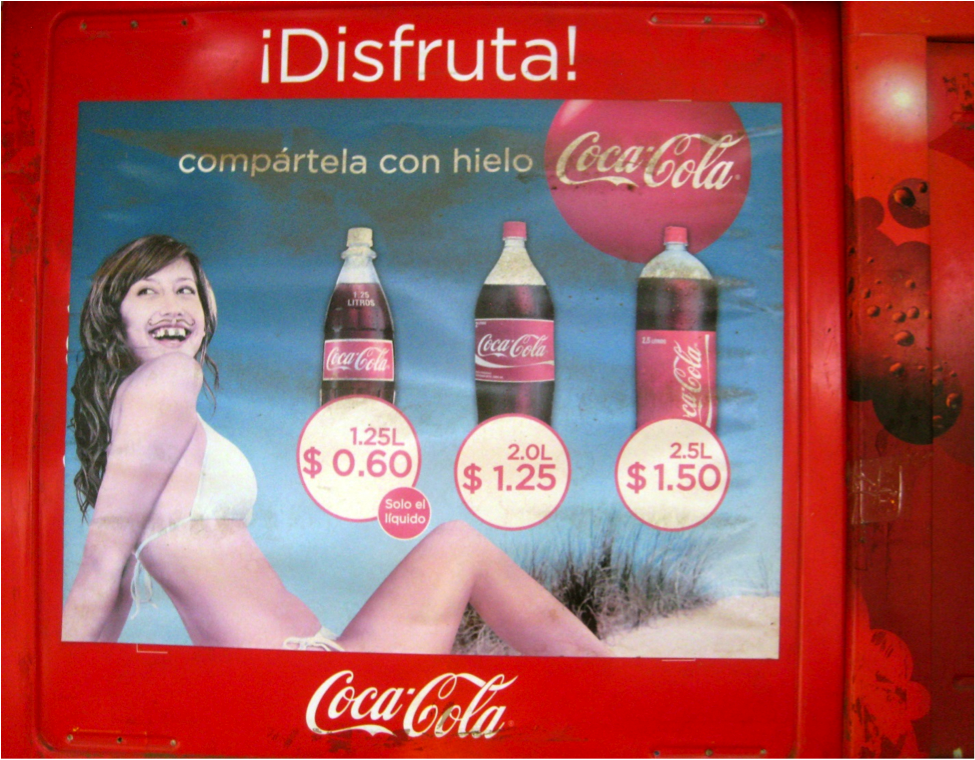

Supplement: Supplementary file 12 — Sex Appeal, combined with Cheap Price theme used in a soda ad in a rural setting. [file 12889_2015_1836_MOESM12_ESM.png]
